# Analysis of Reaction between **α**-Lipoic Acid and 2-Chloro-1-methylquinolinium Tetrafluoroborate Used as a Precolumn Derivatization Technique in Chromatographic Determination of **α**-Lipoic Acid

**DOI:** 10.1155/2015/535387

**Published:** 2015-10-04

**Authors:** Magdalena Godlewska, Angelika Odachowska, Monika Turkowicz, Joanna Karpinska

**Affiliations:** ^1^Faculty of Chemistry, University of Bialystok, Hurtowa 1, 15-399 Bialystok, Poland; ^2^Białystok Provincial Sanitary and Epidemiological Station, Department of Food Products, Food-Contact Articles and Nutrition Research, Legionowa 8, 15-099 Bialystok, Poland

## Abstract

The present study offers results of analysis concerning the course of reaction between reduced *α*-lipoic acid (LA) and 2-chloro-1-methylquinolinium tetrafluoroborate (CMQT). In water environments, the reaction between CMQT and hydrophilic thiols proceeds very rapidly and the resultant products are stable. For the described analysis, optimum reaction conditions, such as concentration of the reducing agent, environment pH, and concentration of the reagent were carefully selected. The spectrophotometric assay was carried out measuring absorbance at λ = 348 nm (i.e., the spectral band of the obtained reaction product). Furthermore, the calibration curve of lipoic acid was registered. It was concluded that the Lambert-Beer law was observed within the range 1–10 *μ*mol L^−1^. Later, the reaction between LA and CMQT was used as precolumn derivatization in a chromatographic determination of the lipoic acid in the range 2.5–50 *μ*mol L^−1^. Practical applicability of the designed methods was evaluated by determining lipoic acid in *Revitanerv* pharmaceutical preparation which contains 300 mg LA in a single capsule. The error of the determination did not exceed 0.5% in relation to the declared value.

## 1. Introduction

**Figure 1:**
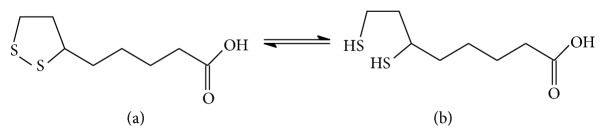
Lipoic acid (LA) (a) and its reduced form dihydrolipoic acid (DHLA) (b).

Lipoic acid (LA, 1,2-dithiolane-3-pentanoic acid, Figure 1(a)), along with its reduced form (dihydrolipoic acid, DHLA, Figure 1(b)), is an important cofactor of mitochondrial enzymes and a natural antioxidant. It is present in both eukaryotic and prokaryotic microorganisms [1] and in all plant and animal cells [2]. In oxidative decarboxylation of pyruvate, *α*-ketoglutarate, branched-chain *α*-keto acids and glycine it acts as a catalyst [3, 4]. During the above processes, lipoic acid undergoes reduction to dihydrolipoic acid. The value of standard redox potential of the dihydrolipoic/lipoic acid couple is equal to −320 mV [3, 5]. Owing to such a high redox potential, the couple serves as an antioxidant for antioxidants [6]. LA is also involved in reduction of such compounds as tocopherol radical, oxidized vitamin C, glutathione, and Q_10_ coenzyme [6]. Lipoic acid is both water- and fat-soluble. For this reason, it is present in blood plasma, cytoplasm, and cell membranes [5, 6]. This property makes it an intermediary agent between lipophilic (tocopherol, Q_10_ coenzyme) and hydrophilic (glutathione) antioxidants [3]. Intracellularly, lipoic acid, together with other antioxidants, acts as a free radical scavenger [3, 6–8]. The acid forms chelate bonds with metal ions [3, 9, 10]. It has been observed that lipoic acid supplementation has beneficial effects in treatment of conditions related to oxidative stress (e.g., atherosclerosis) [6, 11], diabetes [5, 12], cataract, neurodegenerative diseases [13], liver diseases [13], and acquired immunodeficiency diseases [14]. Also, application of lipoic acid in geriatrics has yielded promising results [3, 4, 15]. Notably, the acid and its derivatives have proven to exhibit advantageous effects in treatment of cancers [13, 16].

For humans, the principal source of lipoic acid is food, but the content of the acid in food products varies. In particular, the products of animal origin, especially red meat, contain between 0.25 and 2.36 *μ*g LA g^−1^ [3], whereas foods of plant origin, for example, fresh potatoes, contain between 1.5 and 4.2 *μ*g LA g^−1^ of the substance [17].

Determination of lipoic acid content in clinical samples (i.e., blood, plasma, tissues) or in food products is extremely useful for evaluation of the acid's function in human metabolism. A number of analytic techniques have been developed for that purpose, including mainly gas chromatography (GC) [17, 18], high-performance liquid chromatography (HPLC) with electrochemical detection [19, 20], mass spectrometry [20–22], fluorescent [23, 24] or UV spectroscopy [25, 26], and capillary electrophoresis (CE) [27]. Due to the presence of polar sulfide and carboxyl groups in the LA molecule, the determination with GC technique requires transformation of lipoic acid into nonpolar derivatives, such as S,S-dibenzyl-methyl or S,S-diethoxycarbonyl methyl esters, after its previous reduction with sodium borohydride [17]. Recently, 4-bromomethyl-6,7-dimethoxycoumarin was used as derivatisation reagent for UV and MALDI-TOF detection of lipoic acid [22].

As for the analysis of lipoic acid content by means of high-performance liquid chromatography, employing electrochemical detectors, as well as mass, UV, and fluorescent spectrometers, is carried out. The usage of an electrochemical detector allows for simultaneous denotation of different chemical forms of lipoic acid, including the oxidized and reduced ones. Using spectrophotometric or fluorescent detection, on the other hand, can be quite problematic, because the molecule of the acid does not contain any chromophoric or fluorophoric groups. Therefore, such an analysis requires a prior reaction which would bound the LA molecule with appropriate signaling groups.

**Figure 2:**
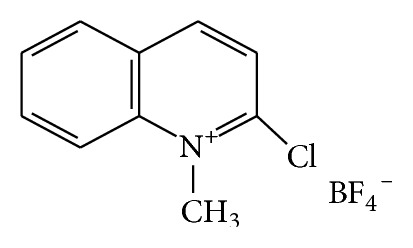
Formulae of 2-chloro-1-methylquinolinium tetrafluoroborate.

The presented study focuses on the course and analytical application of the reaction between reduced form of lipoic acid, dihydrolipoic acid (DHLA) and 2-chloro-1-methylquinolinium tetrafluoroborate (CMQT, Figure 2). The described technique was used for the first time to determine LA content in a pharmaceutical preparation.

## 2. Materials and Methods

### 2.1. Laboratory Equipment

#### 2.1.1. Hitachi U-2800A UV/VIS Spectrophotometer

All spectrophotometric determinations were done using a Hitachi U-2800A spectrophotometer (Japan). The following working settings of the apparatus were applied: scan speed 1200 nm/min and spectral bandwidth (1.5 nm).

The chromatographic system (Thermo Separation) consisted of a 3D Spectra System UV 3000, a low-gradient pump P2000, a vacuum membrane degasser SCM Thermo Separation, and a Rheodyne loop injector (20 *μ*L) and was used for analysis of the lipoic acid derivative solutions. ChromQuest Chromatography Data system software for Windows NT was applied for acquisition and storage of data. The analysis was performed with the use of a Supelco Supelcosil LC-8 HPLC column with the following dimensions: 15 mm length × 4.6 I.D. and 5 *μ*m particle size. Mixture of 5 · 10^−2^ mol L^−1^ pH 3 disodium hydrogen phosphate and acetonitrile in the molar ratio of 35 : 65 was used as mobile phase. The mobile phase flow rate was equal to 1 mL min^−1^, and the chromatograms were monitored at 348 nm.

### 2.2. Reagents

2-Chloro-1-methylquinolinium tetrafluoroborate (CMQT) was prepared directly in the laboratory. For this purpose, the following procedure was employed [28]. In a 50 mL conical flask 1 g of 2-chloro-quinoline and 1 g of 3-methoxytetrafluoroborate were mixed with 1.2 mL of nitromethane. The mixture was stirred until the solid components dissolved, and 4 mL of diethyl ether was added. When the white crystals precipitated, they were drained using a water aspirator and washed twice with 2 mL of diethyl ether. Next, the obtained substance was dried in a desiccator in the presence of CaCl_2_. Thus prepared compound was used to prepare a 10^−2^ mol L^−1^ stock solution by dissolving its weighted measure in MiliQ water. In a similar vein, working solutions with desired concentrations were later obtained by diluting the stock solution with MiliQ water.

Lipoic acid (LA) was produced by Sigma-Aldrich, USA. The stock solution of lipoic acid with the concentration of 10^−2^ mol L^−1^ was obtained by dissolving a proper weighted measure of the substance in methanol. Working solutions were prepared by appropriately diluting the stock solution with MiliQ water.

Sodium borohydride (NaBH_4_) was produced by POCh, Poland. Its stock solution (0.3 mol L^−1^) was prepared by weighing a 0.114 g measure of NaBH_4_ and dissolving it in 10 mL of MiliQ water.

EDTA/NaOH 0.9% NaCl 5 mmol L^−1^ buffer solution was prepared by weighing a 0.731 g measure of EDTA, 0.1 g measure of NaOH, and 0.450 g measure of NaCl and dissolving them in 500 mL of MiliQ water. Solutions with required pH values were obtained by adding appropriate volumes of 1 mol L^−1^ NaOH or HCl.

6% solution of sodium bicarbonate was prepared by weighing a 3 g measure of NaHCO_3_ and dissolving it in 50 mL of MiliQ water.

Stock solution of disodium hydrogen phosphate with the concentration of 5 × 10^−2^ mol L^−1^ and pH 3.00 was prepared by weighing a 3.549 g measure of the solid substance and dissolving it in a 500 mL flask in MiliQ water. The solution's pH 3.00 was achieved by adding 3 mol L^−1^  H_3_PO_4_.

Methanol and acetonitrile (HPLC grade) was produced by Merck, Germany.

*Revitanerv Pharmaceutical Preparation (BLAUFARMA, Poland)*. A single capsule contained microcapsuled *α*-lipoic acid (300 mg), borage seed oil (40% content of *γ*-linolenic acid), gelatin, vitamin E, niacin, pantothenic acid, vitamin B6, vitamin B2, magnesium stearate, silicon dioxide, titanium dioxide, vitamin B1, and selenium.

### 2.3. Experimental Procedures

#### 2.3.1. Spectrophotometric Determination of Lipoic Acid by Means of a Derivatization Reaction

100 *μ*mol L^−1^ lipoic acid solution was introduced into a number of 10 mL test tubes in 0.1, 0.25, 0.5, 0.75, and 1 mL volumes, respectively. After 1 mL of 5 mmol EDTA/NaOH buffer with pH of 9.5 was added into each test tubes. Next 66.8 *μ*L of NaBH_4_ was introduced into each tube to reach final concentration at 0.075 mol L^−1^. The samples were carefully stirred and put in a water bath for 6 minutes at a temperature of 60°C. After the heating stopped, the samples were cooled down to the room temperature. The surplus of unreacted reducing agent was removed by adding 66.8 *μ*L of hydrochloric acid (*c* = 0.5 mol L^−1^), later neutralizing the samples with 85 *μ*L 6% NaHCO_3_. Eventually, the solutions were transferred to 10 mL volumetric flasks.

Next, the volume of 50 *μ*L of CMQT solution with the concentration of 10^−2^ mol L^−1^ was added to prepared DHLA solution. The mixtures were stirred, filled up with MiliQ water to the mark, and carefully stirred once again. Measurements of absorbance were conducted at *λ* = 348 nm 24 minutes after the addition of CMQT, using as a reference the mixture of the same reagents not containing lipoic acid.

#### 2.3.2. Direct Spectrophotometric Technique for Determination of Lipoic Acid

The direct spectrophotometric method for determination of lipoic acid relied on measuring the absorbance of the oxidized form of the acid at *λ* = 210 nm. The procedure was as follows: first, a series of standard LA solutions with concentrations varying between 50 *μ*mol L^−1^ and 500 *μ*mol L^−1^ were prepared. The solutions were obtained by diluting the adequate volumes of the stock solution (*C*_LA_ = 10^−2^ mol L^−1^) with MiliQ water in 10 mL volumetric flasks. Subsequently, the absorbance measurements were conducted at the wavelength of 210 nm (MiliQ water was used as a reference).

#### 2.3.3. Determination of Lipoic Acid in the Pharmaceutical Preparation

The contents of a single capsule were dissolved in a small amount of methanol. The sample was stirred for 20 minutes, and the resultant suspension was filtered. In the next step, the filtrate was transferred to a 100 mL volumetric flask which was then filled up to the mark with methanol. In this way, a stock solution with the concentration of 1.45 · 10^−2^ mol L^−1^ was obtained. During the analysis, a working solution was used, obtained by a hundredfold dilution of the stock solution. Further procedure was as follows: 1 mL of a buffer solution with pH 9.5 was mixed with 412 *μ*L of the working solution obtained from the capsule and 66.8 *μ*L of NaBH_4_ solution (0.3 mol L^−1^). The ensuing preparation was heated for 6 minutes at 60°C. After cooling down, the amounts of 66.8 *μ*L of HCl (0.5 mol L^−1^) and 85 *μ*L of NaHCO_3_ (6%) were added. Next, a 50 *μ*L volume of CMPI solution (10^−2^ mol L^−1^) was introduced; the flask was filled up to the mark with water and carefully stirred. Absorbance of the reaction mixture was measured 24 minutes after stirring at *λ* = 348 nm, using the same mixture of reagents without lipoic acid as reference.

#### 2.3.4. Determination of Lipoic Acid in the Pharmaceutical Preparation Using a Direct Spectrophotometric Technique

The contents of a single capsule were dissolved in a small amount of methanol and the sample was stirred for about 20 minutes. The mixture was later filtered and the filtrate was transferred into a 100 mL volumetric flask which was subsequently filled up to the mark with methanol. Thus obtained solution was tenfold diluted by transferring its 1 mL volume into a 10 mL volumetric flask and filling up the flask to the mark with MiliQ water. Afterwards, 412 *μ*L of this solution was again transferred into a 10 mL flask. The flask was filled up to the mark with MiliQ water and its contents were stirred. Eventually, the absorbance of the ensuing solution was registered at *λ* = 210 nm using water as a reference.

## 3. Results

### 3.1. Preliminary Analysis

**Figure 3:**
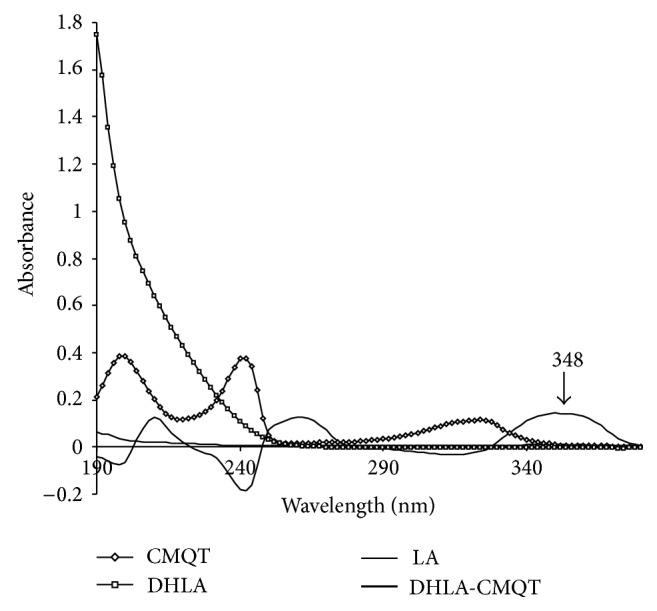
Spectra of lipoic acid (LA), dihydrolipoic acid (DHLA), 2-chloro-1-methylquinolinium tetrafluoroborate (CMQT), and derivatization product (DHLA-CMQT) (concentration of all reagents and compounds = 10^−5^ mol L^−1^; all spectra with exception of derivatization product were recorded against bidistilled water as a blank; spectrum of derivatisation product were recorded against mixture of reagents without DHLA).

Lipoic acid does not contain any chromophoric groups. In the spectrum of an oxidized LA molecule there is a weak band at 330 nm and a more intense nonspecific band at 210 nm (Figure 3).

In the spectrum of the reduced form of LA, the only nonspecific band occurs at 200 nm. As for CMQT, its spectral characteristic is more complex, as can be seen in Figure 3. Specifically, its spectrum features 3 bands: at 200, 240, and 325 nm. The used derivatizing agent selectively reacts with thiol groups, the reaction proceeds rapidly and in a quantitative manner, and the resultant bonding is stable under the conditions of spectrophotometric analysis. The UV spectrum of the CMQT-DHLA reaction product is markedly different from the spectrum of the reagent. In particular, the intensity of the bands at 200 and 240 nm is increased, and new bands appear at 260 and 348 nm, whereas the band at 325 nm diminishes. Due to the intensity of absorbance and practically no interference from the unreacted surplus CMQT, all the measurements described below were carried out at *λ* = 348 nm.

### 3.2. Optimization of the Reaction Conditions

It was established that the intensity of the band at 348 nm was related with the following factors: pH of the lipoic acid reduction environment, temperature and heating time during the reduction, pH of the DHLA-CMQT reaction environment, and NaBH_4_ : LA molar ratio and excess of the used reagent in relation to the amount of reduced lipoic acid. Accordingly, optimization of the reaction conditions was carried out. Selecting different concentrations of the reagents and pH of the environment, the concentration of the analyzed factor was adjusted, keeping the other determinants constant. For the analysis, lipoic acid with the concentration of 10 *μ*mol L^−1^ was used. The exact procedure followed was described above in the equipment and reagents section.  Table 1 contains the resolved parameters of the investigated process. Essentially, it was ascertained that lipoic acid reacted with CMQT in the molar ratio DHLA : CMQT = 1 : 2. For quantitative denotation of lipoic acid in the S-quinolinic derivative form at least a fivefold excess of the reagent should be used.

### 3.3. Spectrophotometric Determination of Lipoic Acid in the Pharmaceutical Preparation

Based on the obtained data the stability constant of the DHLA-CMQT complex was calculated by mole ratio method as *β* = 4.739 × 10^18^. The obtained value shows that the stability of the reaction product makes it eligible for quantitative determination of lipoic acid. Accordingly, ensuring the optimum environment for the DHLA-CMQT bonding, the calibration curve was registered. It was observed that the Lambert-Beer law was fulfilled in the concentration range between 1.0 and 10 *μ*mol L^−1^. The calibration curve was constructed by linear least-square analysis. The limit of detection (LOD) and limit of quantification (LOQ) were calculated based on parameters of calibration curves (LOD = *a* + 3*S*_*y*/*x*_, where *a*-intercept calibration line; $S_{y/x}=\sqrt{{\sum_{i}(y_{i}-{\hat{y}}_{i})}^{2}/(n-2)}$ , ${\hat{y}}_{i}$-value for a given value of *x* readily calculated from the regression equation, LOQ = *a* + 10*S*_*y*/*x*_ [29]). The obtained value of the molar absorption coefficient equaled 1.5 · 10^4^ mol^−1^ · cm^−1^ · L shows the good sensitivity of the determination. Statistical evaluation of the designed spectrophotometric method of LA determination is presented in Table 2. At the same time, a direct method of determination of lipoic acid involving the absorbance measurement of the oxidized form of the acid at 210 nm was devised. It turned out that the direct absorbance measurement allows for determination of lipoic acid in a higher concentration range (i.e. 50–750 *μ*mol L^−1^) with higher LOD and LOQ values. The results are provided in Table 2.

For the sake of practical assessment, the discussed spectrophotometric methods were used to determine lipoic acid in the* Revitanerv* preparation whose declared LA content equals 300 mg per capsule.  Table 3 provides the relevant figures. The data gathered in Table 3 proved the practical usefulness of the developed derivatization procedure. The obtained error of determination is low which testified the good accuracy of the elaborated spectrophotometric method based on derivatisation reaction and good agreement between both spectrophotometric methods is observed.

### 3.4. Application of the DHLA-CMQT Derivatization for Chromatographic Determination of Lipoic Acid

During the investigation of the DHLA-CMQT reaction it was observed that the obtained product was stable. Therefore, it was decided to employ the studied reaction as precolumn derivatization of lipoic acid in a liquid chromatography technique. RP-8 column was used for the purpose, to which 20 *μ*L of the postreaction mixture was transferred. A number of solvents and their mixtures were analyzed (trichloroacetic acid *c* = 0.05 mol L^−1^ : acetonitrile = 95 : 5, methanol : water = 50 : 50, methanol : trichloroacetic acid *c* = 0.05 mol L^−1^ = 10 : 90, acetonitrile : water = 80 : 20, acetonitrile : Na_2_HPO_4_ *c* = 0.05 mol L^−1^ pH 3, the molar ratio varied in the range 80 : 20–20 : 80). The most satisfactory separation was achieved in the case of acetonitrile : Na_2_HPO_4_ *c* = 0.05 mol L^−1^ pH 3 mixed in the volumetric ratio of 35 : 65. Also, it was noted that the retention time of the S-quinolinic derivative of dihydrolipoic acid in the given conditions equaled 3.222 ± 0.001 minutes. Ensuring the optimum conditions of derivatization and preserving the previously established chromatographic process configuration a series of chromatograms were registered for LA solutions with variable concentrations. It was observed that linearity was achieved for the LA concentration range from 2.5 *μ*mol L^−1^ to 50 *μ*mol L^−1^. Statistical evaluation of the devised chromatographic method is presented in Table 2.

It is worth emphasizing that our results are comparable with those obtained by capillary liquid chromatography coupled with ultraviolet detection [22]. The range of detection of proposed HPLC-UV method is 2.5–50 *μ*mol L^−1^ with the LOD = 0.88 pg/injection and LOQ = 2.67 pg/injection which are on the same level as those recently published [22] but worse than in method with fluorescence detection [17, 23, 24]. The proposed procedure was compared with other methods proposed for LA analysis (Table 4).

The comparison shows that the proposed method is superior in terms of its sensitivity and precision. Additionally, it is worth emphasizing that such good parameters were obtained using conventional analytical chromatographic system with DAD detector.

Practical usability of the method was verified by determining the content of lipoic acid in the* Revitanerv* preparation. The results are provided in Table 3. The obtained results testified the good accuracy of the proposed procedure. The error of assay does not exceed 0.5% and good agreement with declared contents of LA is observed.

## 4. Conclusion

The presented study discusses findings related to the investigation of lipoic acid derivatization with the use of 2-chloro-1-methylquinolinium tetrafluoroborate (CMQT). Among others, the experiments allowed concluding that obtaining S-quinolinic derivatives of lipoic acid requires prior reduction of the acid. The product of the reduction is characterized by sufficient stability which renders the reaction applicable for use in spectrophotometric and chromatographic determination of lipoic acid. It is true that introducing derivatization to the determination procedure requires additional effort. The undertaking, however, brings tangible profits. The spectrophotometric method which employs derivatization makes it possible to determine lipoic acid in a lower concentration range in comparison to the direct absorbance measurement techniques. Also, it is marked by lower limits of detection and quantitation. Taking advantage of the analyzed reaction as a precolumn derivatization technique signals a possibility of employing the described process for quantitative determination of lipoic acid in complex biological samples. The presented results showed that the method based on CMQT-DHLA reaction is characterised by sufficient sensitivity which allows using it for direct determination of LA in biological samples [20] without preconcentration of the analyte. The application of reaction-CMQT-LA in chromatographic analysis enables direct assaying of LA in the presence of others low-molecular-mass thiols and their disulfides in biological samples [26, 28].

## Figures and Tables

**Table 1:** Features of the developed methodology.

Parameter	Studied range	Selected value
Molar ratio LA : NaBH_4_	1 : 25–1 : 100	1 : 50
pH of reduction	7–10	9,5
Temperature of reduction/time of heating	60°C 0–15 min	6 min
pH of medium of reaction DHLA-CMQT	4.5–10	6
Excess of CMQT in ratio to DHLA	1 : 1–1 : 5	1 : 5

**Table 2:** Validation parameters of elaborated methods.

Parameter	Direct spectrophotometric method	Spectrophotometric method based on derivatization reaction	HPLC method based on DHLA-CMQT product as precolumn derivatization reaction
Linearity range/*μ*mol L^−1^	50–750	1.0–10	2.5–50
Equation of calibration curve/*x*-concentration in mol L^−1^	*y* = 1.6 · 10^3^ *x* + 1.6 · 10^−3^	*y* = 1.5 · 10^4^ *x* + 0.4 · 10^−3^	*y* = 4.0 · 10^7^ *x* − 110.75
Correlation coefficient	0.999	0.999	0.997
LOD/*μ*mol L^−1^	1.120	0.258	0.214
LOQ/*μ*mol L^−1^	37	0.784	0.647
RSD/%	0.5	3.0	2.06
SD	0.018	7 · 10^−3^	0.014

**Table 3:** Assay results of the commercial lipoic acid dosage forms; *n* = 5.

	Declared value/mg	Determined/mg	Average error/%
Direct spectrophotometric method		298 ± 3.26	±0.46
Spectrophotometric method based on derivatization reaction	300 mg/capsule	300.66 ± 2.87	±0.23
HPLC method based on DHLA-CMQT product as precolumn derivatization reaction		301.44 ± 2.63	±0.48

**Table 4:** Comparison of the proposed method with elaborated methods of analyzing LA.

Method	Derivatisation agent	Range of determination	LOD	LOQ	Reference
HPLC-FL	N-(1-Pyrene)iodoacetamide N-(1-Pyrenemethyl)iodoacetamide	0.75–120 *μ*mol L^−1^	<3.1 fmol	Not given	[23]
	Ammonium 4-fluoro-2,1,3-benzoxadiazole-7-sulfonate (SBD-F)	0.94–60 *μ*mol L^−1^	0.13 pmol	0.44 pmol	[24]

Capillary LC-UV	4-Bromomethyl-6,7-dimethoxycoumarin	0.1–20 *μ*mol L^−1^	0.03 *μ*mol L^−1^	Not given	[21]
	4-Bromomethyl-6,7-dimethoxycoumarin	0.1–40 *μ*mol L^−1^	5 fmol	Not given	[22]

HPLC-UV	Derivatisation with 1-benzyl-2-chloropyridinium bromide	0.2–50 *μ*mol L^−1^	0.1 *μ*mol L^−1^	0.20 *μ*mol L^−1^	[26]
	Without derivatisation	48.5–2422.7 *μ*mol L^−1^	21.32 *μ*mol L^−1^	81.40 *μ*mol L^−1^	[25]

Proposed HPLC-UV method	2-Chloro-1-methylquinolinum tetrafluoroborate	2.5–50 *μ*mol L^−1^	0.21 *μ*mol L^−1^	0.65 *μ*mol L^−1^	
